# *Mycobacterium tuberculosis* Infection: Control and Treatment

**DOI:** 10.3390/microorganisms11041057

**Published:** 2023-04-18

**Authors:** Elena G. Salina

**Affiliations:** 1 Bach Institute of Biochemistry Research Center of Biotechnology, Russian Academy of Sciences, 119071 Moscow, Russia; elenasalina@yandex.ru; 2 Shemyakin-Ovchinnikov Institute of Bioorganic Chemistry, Russian Academy of Sciences, 117997 Moscow, Russia

Tuberculosis (TB) is the second leading infectious killer after COVID-19, causing 10 million new cases and claiming the lives of more than 1.5 million people every year [1]; furthermore, a growing number of multidrug-resistant TB strains constitute a major health threat. During long co-evolution, *Mycobacterium tuberculosis* has developed a plethora of molecular mechanisms that successfully bypass host immunity by expressing specific factors and regulators, which contribute to the progression of the disease [2]. However, the exact mechanisms that allow *M. tuberculosis* to adapt to challenging microenvironments during infection are still poorly understood, hindering efforts to develop novel strategies to control TB. An additional concern is latent TB, which currently affects about 1.7 billion people, putting them at lifetime risk of developing active disease [3].

Thus, improvements in vaccines and diagnostic testing, as well as continued TB drug discovery, will help to reduce disease severity in infected patients and prevent pathogen transmission in the population, especially in the era of the COVID-19 pandemic, with burdens on healthcare systems remaining very high. This Special Issue ‘Mycobacterium tuberculosis Infection: Control and Treatment’ aims to help fill the knowledge gap between the elucidation of the molecular mechanisms of the pathogen’s survival, its interaction with host immunity, the reduction in the TB treatment course and the increase in its effectiveness.

In particular, Bychenko et al.’s research paper identified MTS1338, a small non-coding RNA, as a possible *M. tuberculosis* virulence factor. MTS1338 was found to confer pathogenic properties on non-pathogenic *M. smegmatis* [4]. Specifically, the overexpression of MTS1338 promotes the survival of *M. smegmatis* in macrophages, inducing the arrest of phagosome maturation and modulating the expression of pro-inflammatory cytokines that are characteristic for pathogenic *M. tuberculosis* upon infection, suggesting the role of MTS1338 as a virulence factor that maintains the residence of *M. tuberculosis* in the host. Shepelkova et al. demonstrated significant changes in the miRNAs spectra found in the serum of TB surgery patients with various forms of post-primary pulmonary TB [5]. The authors proposed miRNAs as possible biomarkers that could determine a degree of lung destruction, the intensity of inflammatory processes and the feasibility of the planned surgery.

Interestingly, when Panaiotov et al. investigated the biodiversity of *M. tuberculosis* in Bulgaria [6], they found that human immigration in the last 10 centuries could have played a limited role in the formation of the pathogen’s genetic biodiversity, because only Euro-American Lineage 4 was found to be widely diffused in this country. Immigrations of Gypsies from the Indian subcontinent in the 10th–12th centuries, Turks from Central Asia in the medieval ages, and Armenians, Russians and Africans in the 20th century to Bulgaria affected *M. tuberculosis* biodiversity, but only with sublineage genotypes within the Lineage 4. The authors hypothesized that these sublineages were more virulent or that the ecological adaptation of introduced *M. tuberculosis* genotypes was the main driving force contributing to the current *M. tuberculosis* genetic biodiversity in Bulgaria.

Lagutkin et al. examined the effect of anti-tuberculosis drug treatment on *M. tuberculosis* genome by the whole genome sequencing of isolates obtained before and after treatment, demonstrating several new drug-resistance-associated genetic polymorphisms [7]. Some of them seem to be associated with decreased drug sensitivity or are drug-resistance-driven. Furthermore, they studied intra-host-scale microevolution during treatment and found some divergent loci, acquiring SNPs and/or inserts in two or more series during therapy. These findings could provide new insights into the drug-resistance mechanisms of *M. tuberculosis*. In Kostyukova et al.’s research paper, a collection of isolates from newly diagnosed patients in Western Siberia was studied [8]. The researchers identified a high incidence of resistant strains in particular, and the proportion of MDR strains was found to be 67.9%, of which 40.4% were pre-XDR and 19.2% were XDR. The spectrum of drug resistance is represented by a diverse combination of anti-TB drugs, including second-line drugs, which significantly narrows the variety of effective treatment and increases the risk of adverse outcomes related to tuberculosis.

All this brings the task of finding original drugs with new targets and a lack of resistance to the fore, as well as the problem of timely and effective vaccination. In their review, Nadolinskaia et al. pointed out that the only licensed vaccine, BCG, is unlikely to provide long-term protection against tuberculosis because it is highly variable in its effectiveness [9]. The development of a new vaccine against tuberculosis is a non-trivial scientific task, due to a number of features of the intracellular lifestyle of *M. tuberculosis*, as well as its ability to manipulate host immunity. However, there are some promising strategies to create a new vaccine (supplementing mycobacterial strains with immunodominant antigens, genetic engineering manipulations directed to altering the ‘host–pathogen’ interaction, etc.) that could replace BCG in the near future and provide greater protection against TB.

Obviously, in the absence of an effective anti-tuberculosis vaccine, the search for original drugs for TB, including latent infection, is of particular importance. Ezquerra-Aznárez et al. pointed out the necessity of finding novel antimicrobial agents with new mechanisms of action active against susceptible and drug-resistant microorganisms [10]. The repurposing of medicines has arisen in recent years as a fast, low-cost and efficient strategy to identify novel biomedical applications for already approved drugs. In particular, some anti-parasitic drugs have been found to additionally demonstrate a certain level of anti-tuberculosis activity. The authors also discussed natural products with a dual activity against parasites and *M. tuberculosis*. Several clinical trials tested antiparasitic drugs in TB patients and experienced difficulties with effective dose and toxicity, consistent with natural differences between TB and parasitic infections. However, via medicinal chemistry approaches, some derivatives of antiparasitic drugs have been successfully modified in TB drugs. Thus, the repurposing of antiparasitic medicines for the treatment of TB deserves attention as a potential contribution to TB drug development. Continuing this topic is the review conducted by Alexandrova et al., who analyze the antimycobacterial activity of nucleoside derivatives and their analogues [11]. Despite the fact that there are currently no clinically used drugs based on nucleosides, several series of nucleoside derivatives and analogues with high antimycobacterial activity have been synthesized, and two specific *M. tuberculosis* enzymes, namely thymidine monophosphate kinase and thymidylate synthase, were identified as their targets. Salina and Makarov’s review examined the dormant state of *M. tuberculosis* and latent TB infection, which substantially impact drug tolerance and TB clinical management due to a significant decrease in the metabolic activity of bacilli, leading to the complexity of both the diagnosis and eradication of the bacilli [12]. Evidently, latent TB infection presents a constant risk of disease reactivation, which is 10% over a lifetime in the general population and 10% per year in the case of those with a compromised immune system [13,14].

Thus, the Special Issue includes articles that will be of interest for a wide audience, including researchers, physicians and clinicians, contributing to combating and overcoming the challenges posed by the oldest human pathogen—*M. tuberculosis*.
